# Combined supplementation with isochlorogenic acid and quercetagetin ameliorates dexamethasone-induced intestinal injury via attenuation of oxidative stress and inflammation

**DOI:** 10.3389/fvets.2026.1797403

**Published:** 2026-04-24

**Authors:** Xiaoning Li, Yilin Feng, Songkang Bai, Pengyu Zhao, Ruihui Dai, Ning Li

**Affiliations:** 1 Department of Science and Engineering, Hebei Agricultural University, Cangzhou, China; 2 Hebei Province Plant Source Animal Health Products Technology Innovation Center, Handan, China; 3 Office of Development and Planning, Hebei Agricultural University, Baoding, China

**Keywords:** antioxidant, immune performance, isochlorogenic acid, microbial diversity, quercetagetin

## Abstract

Oxidative stress is a critical factor impairing intestinal function and homeostasis in broilers, particularly under dexamethasone (DEX)-induced conditions. Isochlorogenic acid (ICA) and quercetagetin (QG) are natural flavonoids with known antioxidant properties, but their combined effects remain unclear. A total of 270 one-day-old Arbor Acres broilers were randomly assigned to three groups: control, DEX-induced injury, and ICA+QG treatment (100 mg/kg each). After 21 days of feeding, oxidative stress was induced by intraperitoneal injection of DEX (3 mg/kg). Serum antioxidant indices, immune parameters, intestinal morphology, and gut microbiota composition were analyzed. Compared with the DEX group, ICA+QG supplementation significantly increased serum superoxide dismutase (SOD) and glutathione (GSH) activities (*P* < 0.05), while enhancing intestinal antioxidant capacity. Immune-related indicators, including interleukin-1β (IL-1β), interleukin-4 (IL-4), immunoglobulin M (IgM), and complement component 3 (C3), were significantly elevated (*P* < 0.05). Intestinal morphology was improved, with increased villus height and villus-to-crypt ratio. Moreover, gut microbiota structure was modulated toward a more balanced composition. Combined ICA and QG supplementation alleviates DEX-induced oxidative stress and intestinal damage by enhancing antioxidant defenses, modulating immune responses, and improving intestinal morphology and microbial balance. This strategy may provide a practical nutritional approach to support intestinal function in broilers under stress conditions.

## 1 Introduction

With the gradual reduction in antibiotic use in animal husbandry and increasing awareness of the limitations of traditional synthetic antioxidants, such as low efficiency and potential toxic side effects (1), the development of safe and effective alternatives has become an important research focus in livestock production. Plant extracts possess several advantages, including easy availability, eco-friendliness, low residue, and minimal environmental pollution, making them promising candidates for application in the livestock industry (2). Through physicochemical extraction methods, bioactive compounds—either single compounds or mixtures—can be extracted from plant roots, leaves, stems, and fruits while preserving their original activity. When incorporated into animal feed, these extracts not only provides essential nutrients but also help prevent disease, enhance antioxidant capacity, and improve growth performance (3, 4).

Oxidative stress occurs when the generation of reactive oxygen species (ROS) exceeds the cellular antioxidant defense capacity, leading to oxidative damage to proteins, lipids, and nucleic acids. Excessive oxidative stress suppresses protein synthesis and promotes protein degradation, thereby impairing cellular function and ultimately contributing to muscle atrophy (5, 6). Elevated glucocorticoid levels are widely recognized as physiological markers of stress and have been associated with disturbances in redox homeostasis in poultry (7). Dexamethasone (DEX), a synthetic glucocorticoid, can increase mitochondrial membrane potential and mitochondrial oxidative activity, thereby enhancing ROS production and cellular oxidative stress. This stimulation of cellular metabolism promotes ATP production and continuous generation of various reactive radical and non-radical species. Consequently, DEX administration is widely used to mimic the adverse effects of elevated corticosterone in broilers, induces excessive free radical production, oxidative damage, reduced antioxidant capacity, impaired growth performance, and compromised immune function (8). Several studies have demonstrated the detrimental effects of DEX-induced oxidative stress and the potential protective effects of dietary bioactive compounds. Oh et al. (9) demonstrated that tofu containing *Tenebrio molitor* larvae alleviated DEX-induced muscle atrophy in C_2_C_12_ myotubes and mice by reducing protein degradation. Sanchez-Aceves et al. (10) reported that DEX exposure induced anxiety-like behaviors in zebrafish, enhanced oxidative stress responses, and decreased acetylcholinesterase activity, suggesting a potential ecological and public health threat associated with environmental DEX contamination. Mahmoud et al. (11) found that black pepper oil exerted protective effects against DEX-induced pancreatic injury in mice by suppressing oxidative stress, elevating endogenous antioxidant levels, and mitigating adverse metabolic alterations such as hyperglycemia and dyslipidemia. In poultry research, the DEX-induced oxidative stress model has been widely validated. For example, Fathi et al. (12) reported that dexamethasone administration successfully induced oxidative stress in broilers, whereas melatonin supplementation significantly improved growth performance, serum biochemical parameters, and alleviated oxidative stress and inflammatory responses. In the present study, dexamethasone was therefore used as a controlled short-term experimental stressor to simulate elevated corticosterone levels caused by environmental stress, disease, or management challenges in commercial broiler production.

Isochlorogenic acid (ICA) is a naturally occurring organic compound and an isomer of chlorogenic acid with an additional caffeoyl group. Due to its relatively stable structure, ICA exhibits notable antibacterial, anti-inflammatory, and antioxidant activities (13). Li et al. (14) reported that isochlorogenic acid C exerts a protective effect against oxidative damage in mouse embryonic fibroblast cells by restoring the activity of the SIRT3-SOD2 pathway. Hou et al. (15) found that isochlorogenic acid had strong anti-inflammatory activity in zebrafish embryo *in vivo* model; Liu et al. (16) found that adding stevia extract (containing active ingredients such as chlorogenic acid) to the diet can improve the antioxidant performance of broilers and promote the intestinal health of broilers. However, most existing studies on ICA have primarily focused on rodent models (17), and investigations in broiler chickens remain limited. Quercetagetin (QG) is a flavonoid alcohol compound found in the residue after the extraction of lutein from marigold. Compared with quercetin, QG contains an additional phenolic hydroxyl group, which enhances its reducing ability and antioxidant potential. Nevertheless, relatively few studies have investigated the antioxidant activity of QG under dexamethasone oxidative stress injury. In actual production, the addition of organic acids to animal feed improves intestinal health, enhance antioxidant enzyme activities and modulate gut microbial community structure, thereby enhancing animal production performance and economic benefits (18). Based on previous studies, ICA and QG exhibit strong anti-inflammatory and antioxidant properties, which can alleviate the damage caused by animal oxidative stress and mitigate oxidative stress-related tissue damage and modulate inflammatory responses. Therefore, this study aimed to investigate the effects of a combination of ICA and QG on the antioxidant capacity, immune performance, intestinal morphology, and gut microbiota of Arbor Acres broilers under a DEX-induced oxidative stress model.

Prior to the formal experiment, a preliminary trial was conducted to determine the optimal supplementation level of quercetagetin. In the preliminary experiment, the initial QG dosage gradient was set at 25, 50, 100, and 200 mg/kg. The results of the preliminary experiment indicated that the QG dosage of 100 mg/kg exerted the most prominent effect on alleviating oxidative stress-induced damage in broilers. Consistently, Liang et al. (19) demonstrated that dietary supplementation with 100 mg/kg QG could significantly enhance the antioxidant capacity of broilers, improve the digestibility of nutrients, and elevate their growth performance.

In addition, Wang et al. (20) revealed that dietary supplementation with 200 mg/kg of isochlorogenic acid (ICA) could effectively improve the antioxidant capacity, immune function, and intestinal microflora composition of weaned piglets. Based on the preliminary experimental results and previous literature, the present study selected a combined supplementation of 100 mg/kg ICA and 100 mg/kg QG. Therefore, the objective of this study was to investigate the effects of dietary supplementation with ICA and QG on antioxidant capacity, immune performance, intestinal morphology, and gut microbiota in Arbor Acres broilers under a dexamethasone-induced oxidative stress model. This study aims to provide new insights into the potential application of ICA and QG as functional feed additives in poultry production.

## 2 Materials and methods

### 2.1 Experimental materials and animals

Two hundred and seventy 1-day-old AA broilers (genetic line: Arbor Acres, commercial meat-type broiler strain) were purchased from Handan Huixiang Agricultural Science and Technology Co., Ltd. The initial average body weight of the broilers was 43 ± 2 g. All birds were confirmed to be healthy— free of clinical signs of disease, exhibiting normal appetite, and showing consistent growth status—after a 3-day acclimation period prior to the start of the formal experiment.

Isochlorogenic acid (total acid content: 55%, verified by HPLC) and quercetagetin (purity ≥ 97%, verified by HPLC), provided by Hebei Chenguang Biotechnology Co., Ltd., were incorporated into the basal diet in powder form. Dexamethasone was obtained from commercial sources. To establish an oxidative stress model, dexamethasone was administered via intraperitoneal injection at a single dose of 3 mg/kg body weight.

### 2.2 Experimental design

#### 2.2.1 Selection and grouping of experimental animals

All the experimental protocols were approved by the Experimental Animal Ethics Committee of Hebei Agricultural University (Number. 2022155; approval date: March 29, 2022).

A total of 270 1-day-old Arbor Acres (AA) broilers with similar body weight and good health status were used in this study. The initial average body weight was 43 ± 2 g. Birds were randomly assigned to three experimental groups using a completely randomized design: a control group (basal diet), an injury group (basal diet with dexamethasone induction at 3 mg/kg body weight), and a combination group (basal diet supplemented with 100 mg/kg isochlorogenic acid and 100 mg/kg quercetagetin). Each group consisted of five replicates with 18 birds per replicate.

#### 2.2.2 Experimental cycle and processing time nodes

The experiment was conducted from July to September 2023 at Handan Chenguang Plant Protein Co., Ltd. Before the initiation of the feeding trial, a 3-day acclimation period was implemented. During this period, all birds were housed under identical environmental conditions, fed the same basal diet, and managed in strict accordance with standardized husbandry protocols. This acclimation period was intended to ensure that the experimental animals fully adapted to the experimental environment, dietary formulation, and management conditions.

The formal experiment commenced on the 4th day post-acclimation. On day 4, broilers were allocated to their respective experimental groups and fed with diets formulated according to the predefined group-specific regimens for a continuous 21-day period (until the broilers reached a final body weight of 480 ± 25 g). On the 18th day of the formal feeding trial, broilers in both the injury group and the combination group were intraperitoneally injected with a single dose of dexamethasone at a concentration of 3 mg/kg of body weight to induce oxidative damage.

#### 2.2.3 Establishment of the injury model

The dexamethasone-induced oxidative stress model was used to simulate elevated corticosterone levels commonly observed in commercial poultry production under environmental stress or disease conditions. Although DEX administration may elicit certain physiological stress responses, it serves here as a controlled and short-term experimental stressor to scientifically evaluate the protective effects of combined isochlorogenic acid and quercetagetin extract against oxidative damage. In a preliminary experiment, three injection regimens were tested: three injections of 1 mg/kg, a single injection of 3 mg/kg, and a single injection of 8 mg/kg dexamethasone. The 8 mg/kg dose resulted in complete mortality by day 21, whereas the efficacy of three 1 mg/kg injections was inferior to that of the single 3 mg/kg treatment. Therefore, a single intraperitoneal injection at 3 mg/kg dexamethasone was selected for the formal trial.

### 2.3 Feeding management

Before the birds were housed, the poultry facility and equipment were disinfected with sodium hypochlorite. Physical biosecurity measures were implemented to reduce the risk of pathogen introduction, preventing intrusion by animals carrying infectious agents, especially wild birds, rodents, and cats. During the rearing period, broilers had ad libitum access to feed and water and received routine vaccinations.

The temperature of the poultry house was maintained at 35 °C during the first week and gradually decreased by 2–3 °C per week until reaching 26 °C. Relative humidity was maintained at 60–80%. Broilers were housed in three-tier stepped cages and provided with artificial lighting for 16 h/day during the first 10 days and 20 h/day thereafter. All groups were allowed free access to feed and water during the entire experimental period.

### 2.4 Sample collection

On day 21, body weight was recorded. Four broilers with body weights close to the replicate average were randomly selected from each replicate for sampling. Blood samples were collected from the jugular vein, allowed to clot for 2 h, and centrifuged at 3000 r/min for 15 min to obtain serum, which was stored at −20 °C for subsequent analysis of antioxidant and immune parameters.

After blood collection, birds were euthanized by jugular exsanguination. Liver, spleen, small intestine, and intestinal mucosa samples were collected, immediately frozen at −80 °C, and used for antioxidant assays. Liver homogenates were prepared with a homogenizer prior to measurement. All procedures were performed under standardized laboratory conditions to ensure consistency and reproducibility of the results.

### 2.5 Determination indexes and methods

#### 2.5.1 Antioxidant index

The activities of superoxide dismutase (SOD), glutathione (GSH), catalase (CAT), and malondialdehyde (MDA) in serum and intestinal mucosa were determined using enzyme-linked immunosorbent assay (ELISA) kits (Soleberg Bioscience and Technology Ltd.) according to the manufacturer’s instructions.

#### 2.5.2 Immune indicators

Serum immune parameters were measured using commercial ELISA kits purchased from Shanghai Enzyme-linked Biotechnology Co., Ltd. The measured indicators included immunoglobulin A (IgA), immunoglobulin G (IgG), immunoglobulin M (IgM), complement component 3 (C3), complement component 4 (C4), interleukin-1β (IL-1β), interleukin-4 (IL-4), interleukin-10 (IL-10), and tumor necrosis factor-α (TNF-α).

#### 2.5.3 Metrics of intestinal observation

Intestinal tissue samples were collected from the duodenum, jejunum, and ileum of broilers. The intestinal lumen was thoroughly rinsed with physiological saline to remove digesta, after which the tissues were immediately fixed in 4% paraformaldehyde solution. Following fixation, the samples were trimmed into approximately 1-mm segments and processed for histological examination. The tissues underwent a series of standard procedures, including dehydration, embedding in paraffin, sectioning, and staining with hematoxylin and eosin (H&E), to prepare paraffin sections for morphological analysis.

Whole-slide images were acquired using a PANNORAMIC digital slide scanner (3DHISTECH, Hungary) equipped with a 20 × objective lens under uniform illumination. Images were visualized using CaseViewer 2.4 software (3DHISTECH), and morphometric analysis was performed using Image-Pro Plus 6.0 (Media Cybernetics, USA). Two sections per intestinal segment were prepared for each bird. All measurements were conducted by an observer blinded to the treatment groups to minimize bias. Five intact and well-oriented villi were randomly selected in each section to measure villus length and corresponding crypt depth, and the villus length-to-crypt depth ratio (V/C) was subsequently calculated.

#### 2.5.4 Determination of the gut microbiota

Cecal content samples from broilers were collected and immediately transported on dry ice to Biomarker Technologies Co., Ltd. (Beijing, China) for microbiota profiling. Total microbial genomic DNA was extracted using the E.Z.N.A.® Soil DNA Kit according to the manufacturer’s instructions. DNA integrity was assessed by 1% agarose gel electrophoresis, and concentration and purity were measured using a NanoDrop 2000 spectrophotometer.

The V3–V4 hypervariable regions of the bacterial 16S rDNA gene were amplified using the universal primer pair 338F (5’-ACTCC TACGGGAGGCAGCAG-3′) and 806R (5’-GGACTACHVGGGT WTCTAAT-3′). Polymerase chain reactions (PCR) were performed in triplicate using high-fidelity DNA polymerase to minimize amplification bias. Thermal cycling conditions included an initial denaturation at 95 °C for 3 min, followed by 27 cycles of 95 °C for 30 s, 55 °C for 30 s, and 72 °C for 45 s, with a final extension at 72 °C for 10 min. Purified amplicons were pooled at equimolar concentrations and sequenced using the Illumina MiSeq PE300 platform (2 × 300 bp).

Raw sequencing data were processed using the QIIME2 (version 2022.2). After demultiplexing and quality filtering, denoising was performed using the DADA2 plugin, which includes error correction, chimera removal, and inference of amplicon sequence variants (ASVs). Taxonomic assignment of representative ASVs was performed using a pre-trained Naïve Bayes classifier against the SILVA reference database (version 138).

Microbial diversity was comprehensively evaluated using alpha and beta diversity analyses. Alpha diversity was assessed using the Shannon index to estimate microbial richness and evenness, and rarefaction curves were generated to evaluate sequencing depth adequacy. Beta diversity, which describes the compositional dissimilarity of microbial communities between different groups, was analyzed using QIIME software to obtain microbial community composition matrices first, followed by data standardization to eliminate the influence of different sequencing depths among samples. Subsequently, principal component analysis (PCA) was performed based on standardized Euclidean distance matrices using R software: the original community composition data were transformed into a few principal components (PCs) that could explain the maximum variance of the original data, and the first two principal components (PC1 and PC2) with the highest explanatory power were selected for visualization. This PCA plot intuitively displays the clustering patterns and compositional differences of microbial communities across groups, where samples with similar community compositions cluster closely together.

At the phylum and genus taxonomic levels, differences in microbial relative abundance among the control, injury, and combination groups were statistically analyzed using one-way analysis of variance (ANOVA) followed by Duncan’s multiple comparison test. Linear discriminant analysis effect size (LEfSe) was further conducted with an LDA score threshold of 2.0 to identify significantly discriminative taxa (*p* < 0.05).

#### 2.5.5 TMT quantitative proteomic analysis

Tandem Mass Tags (TMT)-based quantitative proteomic analysis was performed by BGI Genomics Co., Ltd. using isobaric labeling combined with liquid chromatography–tandem mass spectrometry (LC–MS/MS).

Frozen intestinal mucosa samples were homogenized in lysis buffer (8 M urea, 1% protease inhibitor cocktail, 1% phosphatase inhibitor), sonicated on ice, and centrifuged at 12,000 × g for 15 min at 4 °C. The supernatants were collected, and protein concentrations were determined using a BCA assay kit. Protein quality was assessed by SDS–PAGE.

For digestion, 100 μg protein per sample was reduced with 5 mM dithiothreitol (56 °C, 30 min) and alkylated with 11 mM iodoacetamide (room temperature, 15 min, dark). The urea concentration was diluted to <2 M with 100 mM triethylammonium bicarbonate, followed by overnight digestion at 37 °C with sequencing-grade modified trypsin at a 1:50 (w/w) ratio and a second digestion at 1:100 for 4 h.

Peptides were labeled using the TMT 6-plex or 10-plex isobaric label reagents according to the manufacturer’s instructions. After incubation for 1 h at room temperature, reactions were quenched with 5% hydroxylamine. Labeled peptides were pooled, desalted using C18 cartridges, and vacuum-dried.

Peptides were separated using an EASY-nLC 1,200 UHPLC system with a gradient of 0.1% formic acid in 80% acetonitrile and analyzed on a Q Exactive™ HF-X Orbitrap mass spectrometer in data-dependent acquisition mode. MS1 scans (m/z 350–1,600) were acquired at 60,000 resolution, and MS/MS scans were acquired at 15,000 resolution with a normalized collision energy of 32. Dynamic exclusion was set to 30 s, and AGC targets were 3 × 10^6^ (MS1) and 1 × 10^5^ (MS2).

Trypsin/P was specified as the cleavage enzyme with up to two missed cleavages. Carbamidomethylation (C) was set as a fixed modification, while oxidation (M) and protein N-terminal acetylation were defined as variable modifications. TMT 6/10-plex reporter ions were used for quantification. The false discovery rate (FDR) was controlled at <1% at both peptide and protein levels.

Differentially expressed proteins were defined by a fold change >1.2 or <1/1.2 and *p* < 0.05 based on one-way ANOVA. Functional enrichment analysis was performed based on Gene Ontology (GO) and Kyoto Encyclopedia of Genes and Genomes (KEGG) databases.

#### 2.5.6 Data statistics and analysis

After normalization of all variables to percentages to reduce variability arising from differences in absolute values and to facilitate visualization, statistical analyses were conducted using SPSS 26.0. One-way ANOVA was applied, followed by Duncan’s multiple range test when treatment effects were significant. Data are expressed as mean ± SD, and statistical significance was defined as *p* < 0.05. Figures were generated using Origin Pro 2018 software.

## 3 Results

### 3.1 Effects of isochlorogenic acid combination on antioxidant properties of serum and intestinal mucosa in broilers

**Figure 1:**
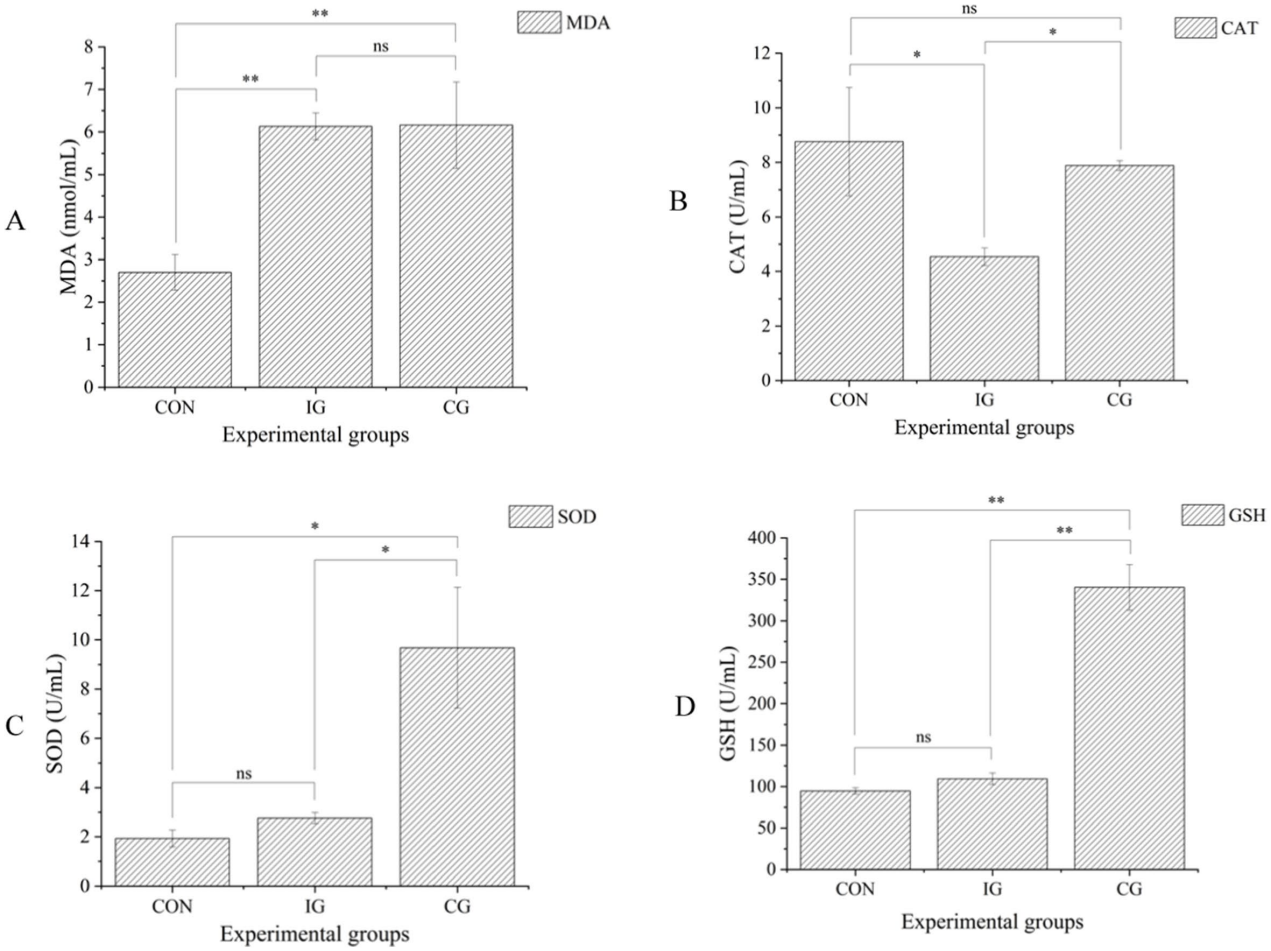
The activity levels of malondialdehyde (MDA) **(A)**, catalase (CAT) **(B)**, superoxide dismutase (SOD) **(C)**, and glutathione (GSH) **(D)** in the serum of broilers from the control group (CON), injury group (IG), and combination group (CG). Data are expressed as mean ± SD (*n* = 5). ns indicates no significant difference; *p < 0.05, **p < 0.01, ***p < 0.001.

Compared with the control group, the serum MDA content of broilers in the injury group was significantly increased (*p* < 0.01; Figure 1), whereas CAT activity was significantly decreased (*p* < 0.05). No significant differences were observed in SOD and GSH activities between the injury group and the control groups (*p* > 0.05). Relative to the injury group, the combination group exhibited significantly higher serum CAT, SOD, and GSH activities (*p* < 0.05). Additionally, compared with the control group, the combination group showed significantly increased serum MDA, SOD, and GSH levels (*p* < 0.05).

**Figure 2:**
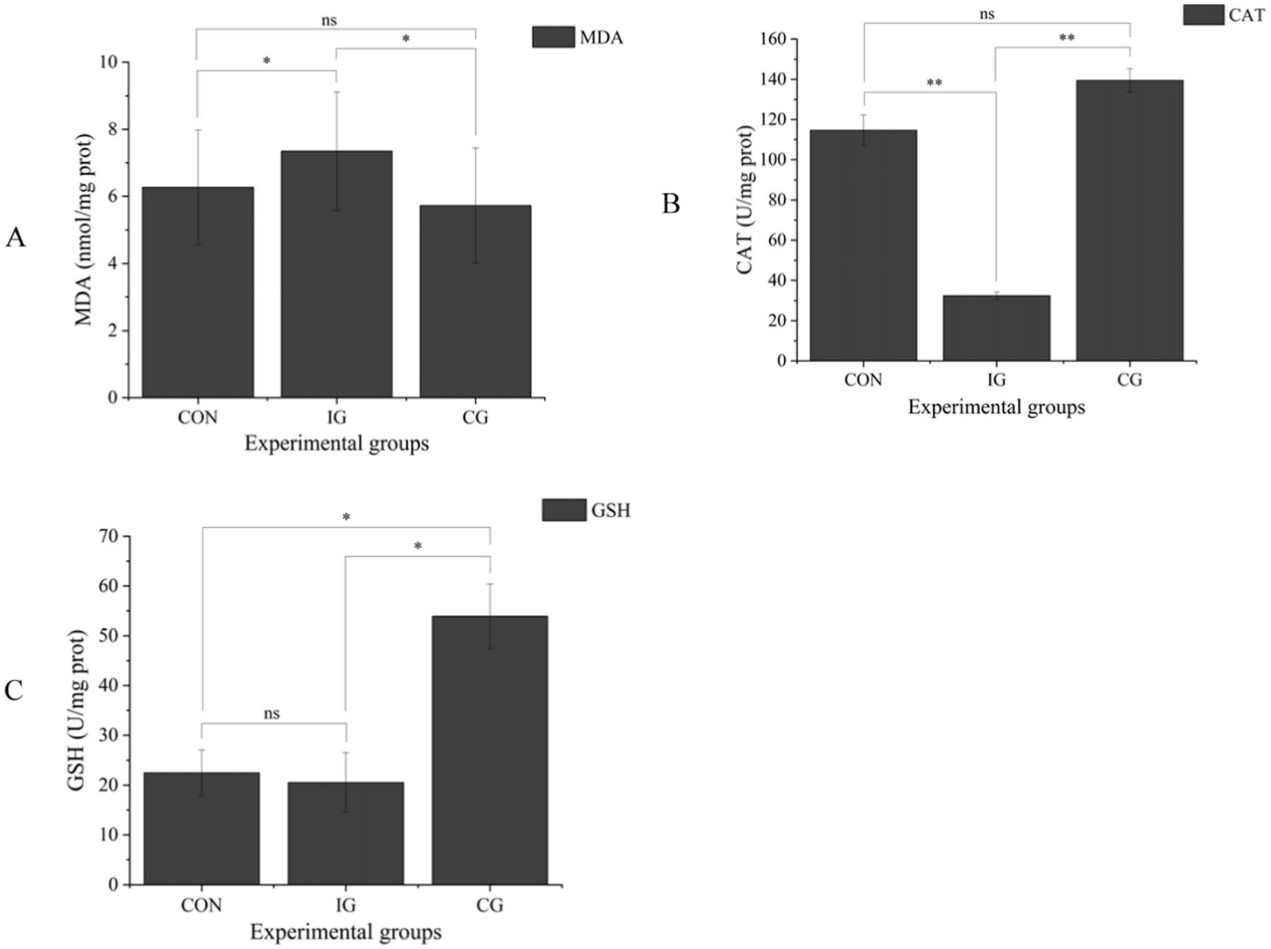
The MDA levels **(A)**, CAT activities **(B)**, and GSH activities **(C)** in the intestinal mucosa of broilers in the CON, IG, and CG groups. Data are presented as mean ± SD (*n* = 5). ns indicates no significant difference; *p < 0.05, **p < 0.01, ***p < 0.001.

In the intestinal mucosa, the injury group showed a significant increase in MDA content (*p* < 0.05; Figure 2) and a highly significant decrease in CAT activity (*p* < 0.01), while GSH activity remained unchanged (*p* > 0.05) compared with the control group. Compared with the injury group, the combination group showed a significant reduction in MDA content (*p* < 0.05) and significant increases in CAT and GSH activities (*p* < 0.05). When compared with the control group, MDA content was lower and CAT activity was higher in the combination group, although these differences were not statistically significant (*p* > 0.05), whereas GSH activity was significantly increased (*p* < 0.05).

### 3.2 Effect of isochlorogenic acid combination on serum immune performance of broilers

**Figure 3:**
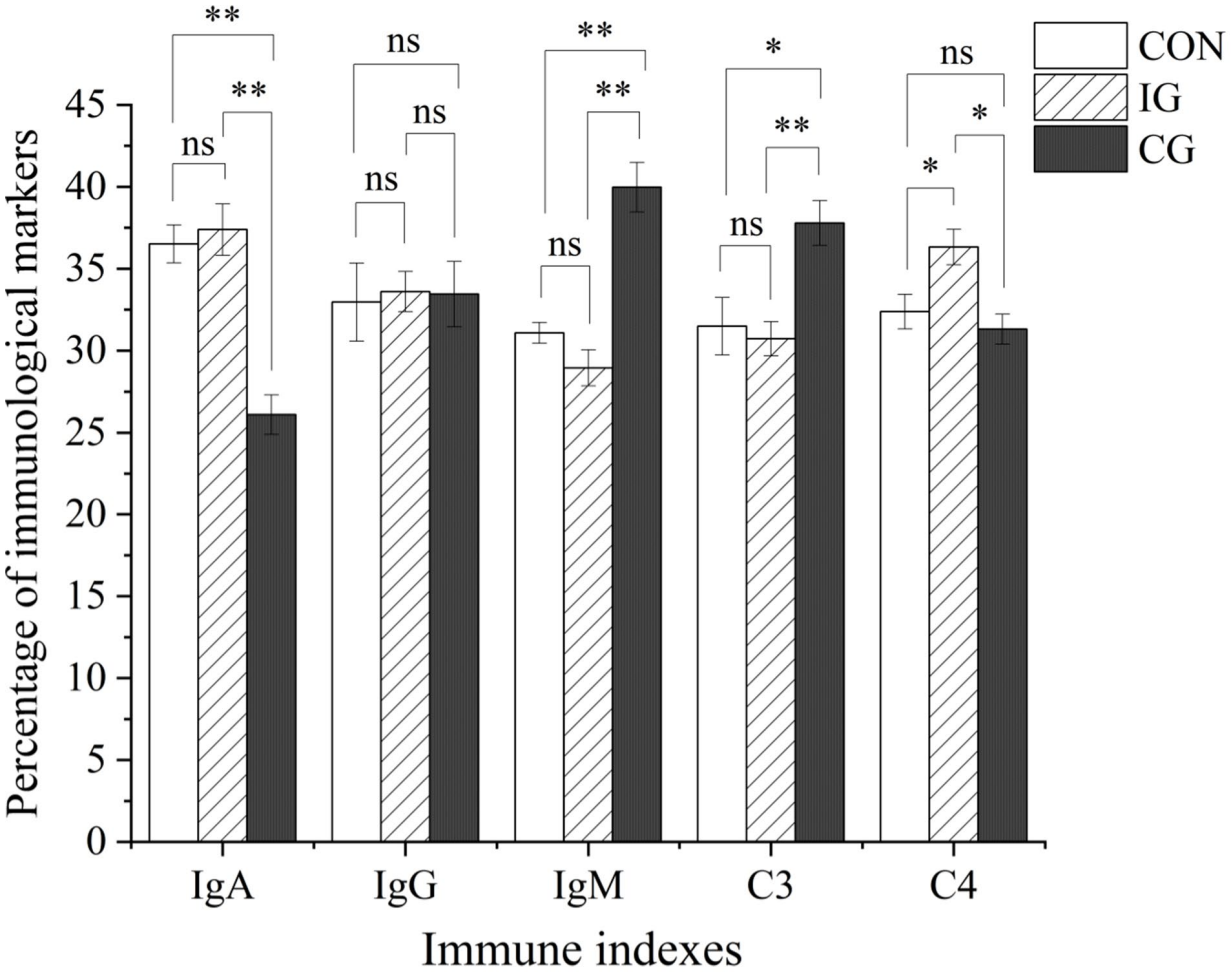
Effects of isochlorogenic acid and quercetagetin combination on serum immunoglobulin and complement levels in broilers. Serum concentrations of IgA, IgG, IgM, C3, and C4 in broilers from CON, IG, and CG groups. Data are presented as mean ± SD (*n* = 5). ns indicates no significant difference; *p < 0.05, **p < 0.01, ***p < 0.001.

For immune parameters, serum IgM and C3 levels in the injury group were lower than those in the control group, although the differences were not significant (*p* > 0.05; Figure 3). In contrast, IgA and IgG levels were increased without statistical significance (*p* > 0.05), while C4 was significantly elevated (*p* < 0.05). Compared with the injury group, the combination group showed significantly increased IgM (*p* < 0.05) and markedly elevated C3 (*p* < 0.01), along with significantly reduced IgA and C4 levels (*p* < 0.05), whereas IgG remained unchanged (*p* > 0.05). Relative to the control group, the serum IgM content in the combination group increased extremely significantly (*p* < 0.01), and the C3 content increased significantly (*p* < 0.05), the IgA content decreased extremely significantly (*p* < 0.01), and no significant differences in IgG and C4 (*p* > 0.05).

**Figure 4:**
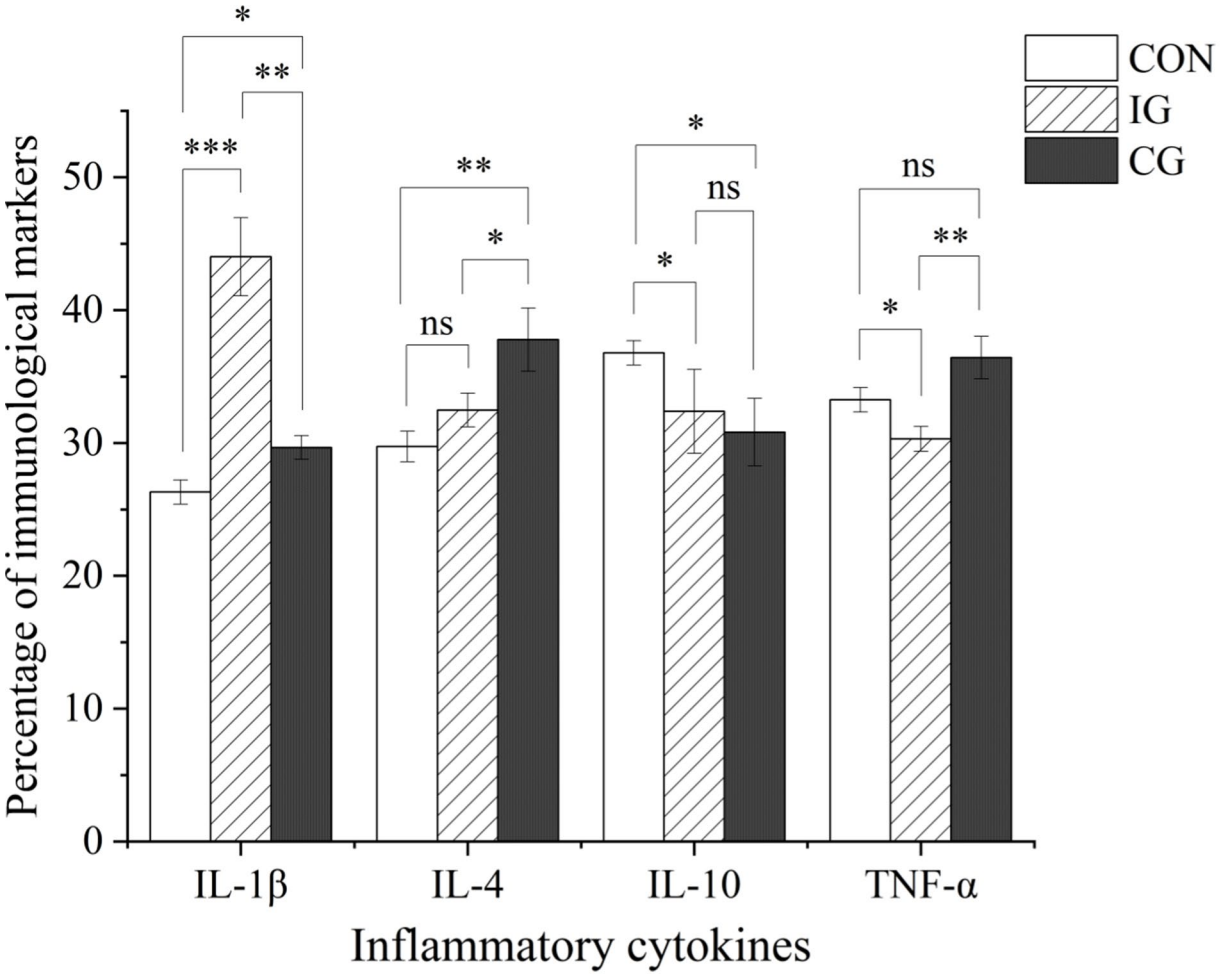
Effects of isochlorogenic acid and quercetagetin combination on serum cytokine levels in broilers. Serum levels of IL-1β, IL-4, IL-10, and TNF-α in broilers from CON, IG, and CG groups. Data are presented as mean ± SD (*n* = 5). ns indicates no significant difference; *p < 0.05, **p < 0.01, ***p < 0.001.

Regarding inflammatory cytokines, the injury group showed a significantly lower serum IL-10 level compared with the control group (*p* < 0.05; Figure 4). Compared with the injury group, the serum IL-4 and TNF-α contents of broilers in the combination group were significantly increased (*p* < 0.05). Relative to the control group, the serum IL-1β and IL-4 levels of broilers in the combination group were significantly increased (*p* < 0.05), TNF-α showed an increasing trend without statistical significance (*p* > 0.05), and IL-10 was significantly decreased (*p* < 0.05).

### 3.3 Effects of isochlorogenic acid combination on intestinal tissue of broilers

**Figure 5:**
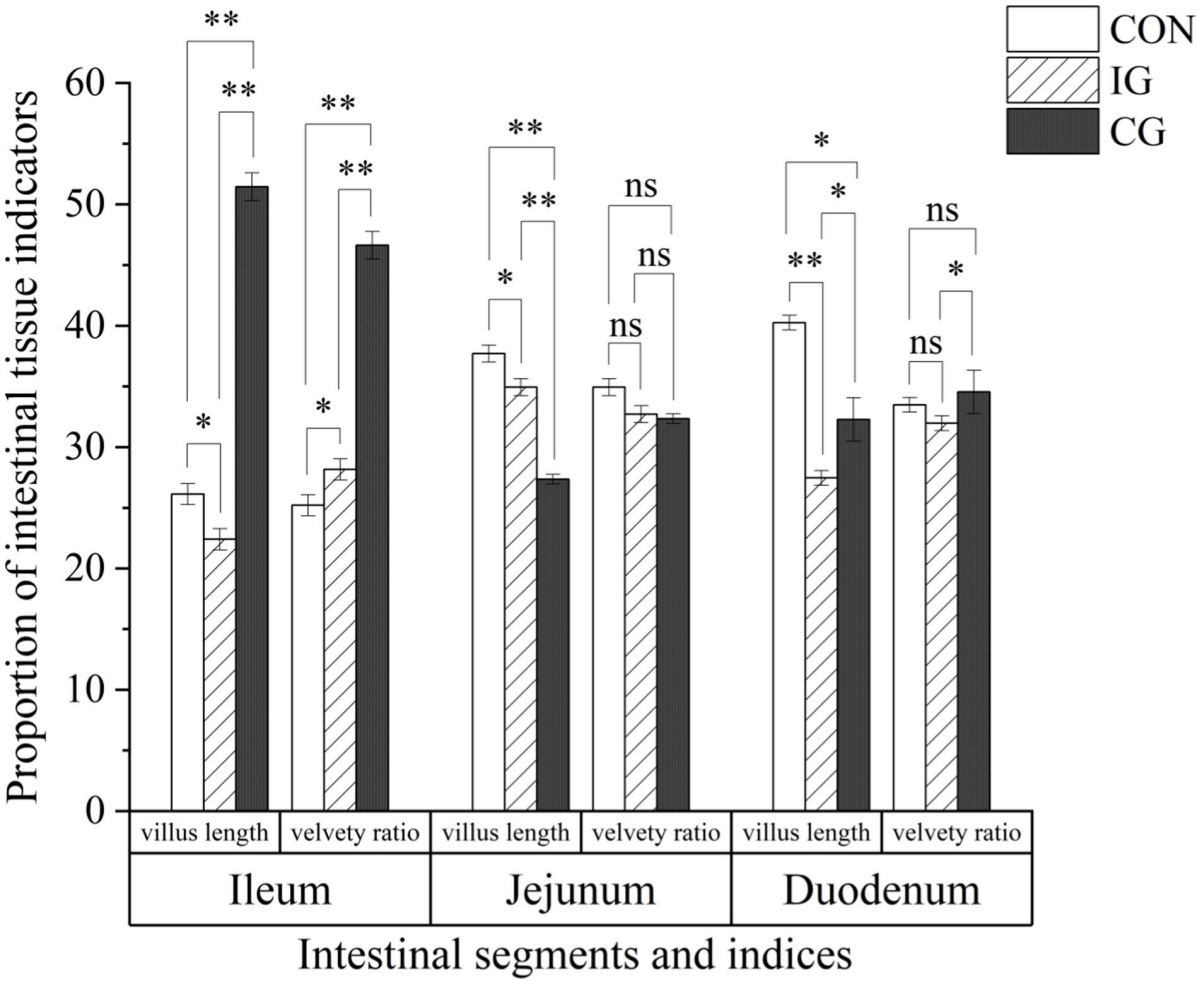
Effects of isochlorogenic acid and quercetagetin combination on intestinal morphology of broilers. Villus length and velvety ratio in the ileum, jejunum, and duodenum of 21-day-old broilers from CON, IG, and CG groups. ns indicates no significant difference; *p < 0.05, **p < 0.01, ***p < 0.001.

With respect to intestinal morphology, compared with the control group, villus length in the ileum and jejunum of broilers of the injury group was significantly reduced (*p* < 0.05; Figure 5), while that in the duodenum was markedly reduced (*p* < 0.01). The velvety ratio in the jejunum and duodenum decreased in the injury group, although the differences were not significant (*p* > 0.05). Compared with the injury group, the villus length in the ileum of broilers in the combination group was extremely significantly increased (*p* < 0.01), that in the duodenum was significantly increased (*p* < 0.05), the velvety ratio in the ileum was extremely significantly increased (*p* < 0.01), and that in the duodenum was significantly increased (*p* < 0.05). Compared with the control group, both villus length and velvety ratio in the ileum were significantly higher in the combination group (*p* < 0.01).

**Figure 6:**
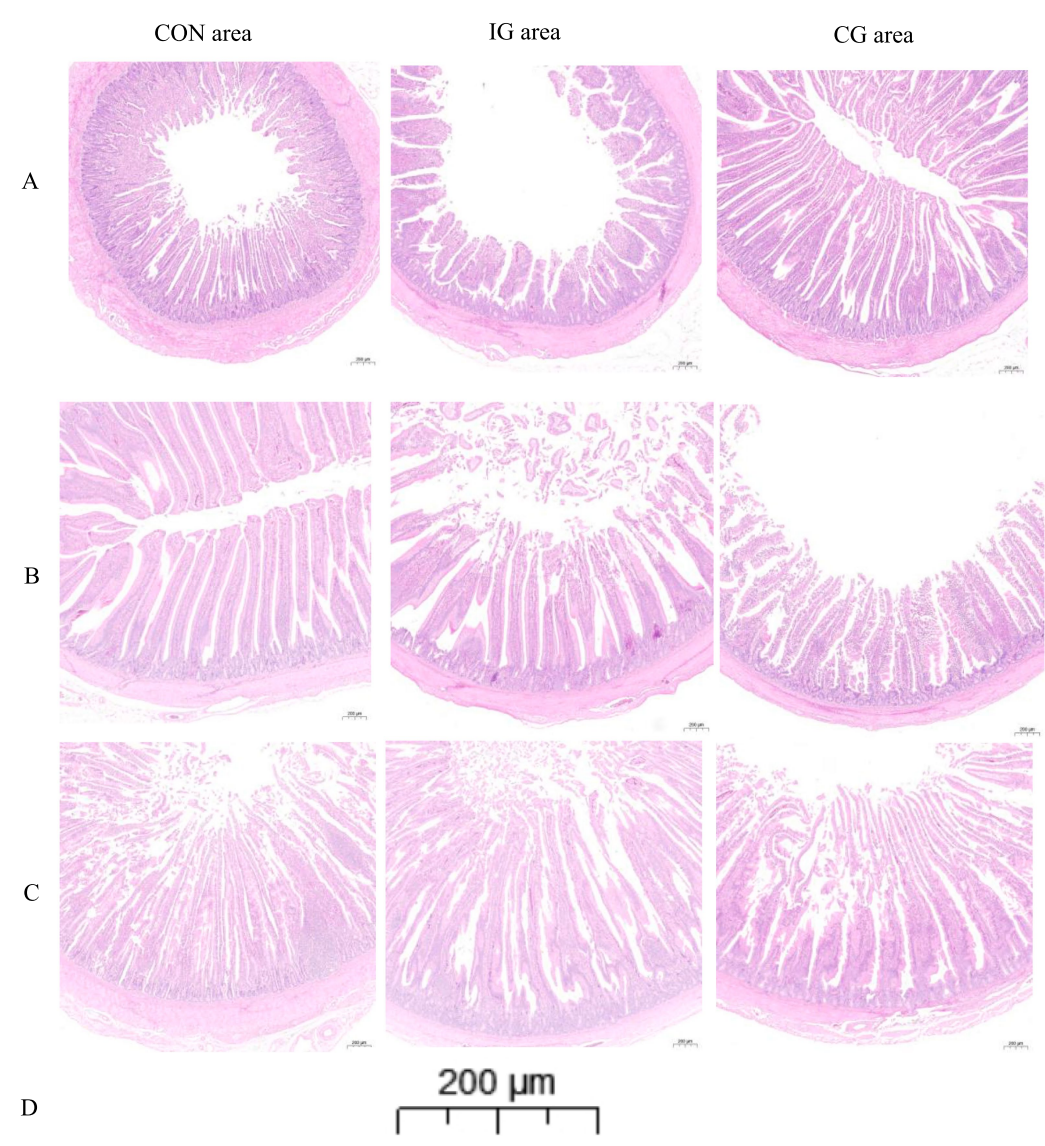
Representative hematoxylin eosin stained sections of **(A)** ileum, **(B)** jejunum, and **(C)** duodenum from CON, IG, and CG groups. Images were acquired using a 3DHISTECH PANNORAMIC whole-slide scanner with a 20× objective (scale bar **(D)** = 200 μm).

Figure 6A depicts the morphological structure of the 21-day-old ileum in different treatment groups. In the control group, villi appeared relatively thick and short, with evident exfoliation and damage, and a low villus density per unit area. In the injury group, villi were predominantly thick and short, with severe exfoliation, structural damage, reduced villus density, and signs of impaired development. In contrast, the combination group exhibited elongated and relatively slender villi, with a more uniform structure and higher villus density, indicating improved villus development. Compared with the control group, the injury group showed potential pathological status, whereas the combination group exhibited smaller crypt depth, a higher villus crypt ratio, weaker potential pathological status, and improved villus architecture.

Figure 6B depicts the morphological structure of the 21-day-old jejunum across different treatment groups. In the control group, the jejunal villi appeared elongated, thick, and columnar, with a reduced villus density per unit area. In the injury group, most villi were long and relatively thick, with a low number of intestinal villi per unit area and visible villus defects. In the combination group, villi were longer and thinner, more orderly arranged, and exhibited higher villus density per unit area. Moreover, differences in the villus crypt ratio among the three groups were minimal, indicating that the isochlorogenic acid combination may mitigate jejunal villus defects.

Figure 6C depicts the morphological structure of the 21-day-old duodenum across different treatment groups. Both the control and combination groups exhibited finger-like villi with slender morphology; however, villi in the combination group were more regularly arranged. In the injury group, villi were thick and relatively short, with a columnar structure and evident exfoliation and damage in some areas. Compared with the control group, the combination group showed a higher villus crypt ratio, indicating that isochlorogenic acid combination treatment may enhance the absorptive function of the duodenum.

### 3.4 Differential protein functional enrichment analysis

#### 3.4.1 Differential protein screening

**Figure 7:**
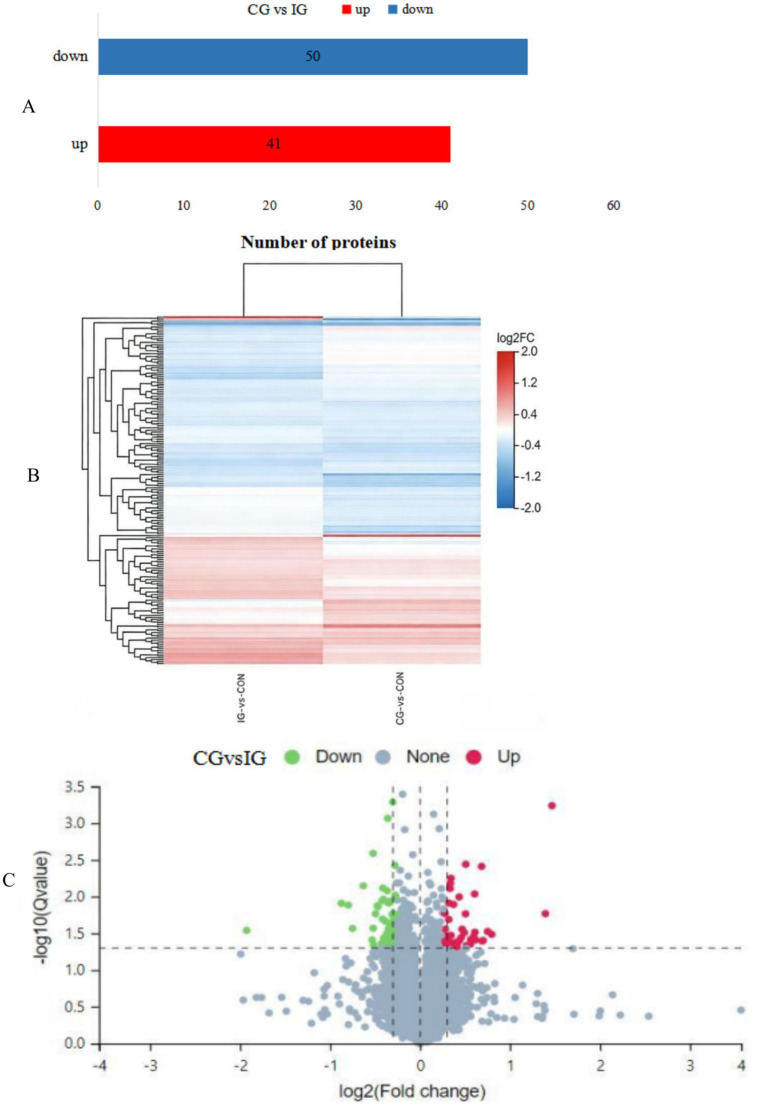
Differentially expressed proteins identified by TMT-based quantitative proteomics. **(A)** Histogram of differentially expressed proteins: CG vs. IG; **(B)** Heatmap of protein expression patterns: IG vs. CON and CG vs. CON; and **(C)** Volcano plot comparing the CG vs. IG.

A one-way ANOVA was performed on the relative quantitative values of each protein. As shown in the volcano plot comparing the combination group with the injury group (Figure 7C), a *p*-value < 0.05 was used as the significance threshold. The ratio of relative quantitative values between groups was taken as the fold change (FC), with FC > 1.2 set as the threshold for significant upregulation and FC < 1/1.2 as the threshold for significant downregulation. Proteins meeting both the significance and differential expression criteria were identified as differentially expressed proteins (DEPs). The final set of DEPs is presented in Figure 7A, where red indicates significantly upregulated proteins and blue indicates significantly downregulated proteins. Compared with the control group, the combination group exhibited 41 significantly upregulated proteins and 50 significantly downregulated proteins. Hierarchical cluster analysis of DEPs was performed, and the results are presented as a heatmap (Figure 7B). Comparing the combination vs. control and injury vs. control groups, it was observed that the combination group exhibited good intra-group reproducibility, while significant inter-group differences were detected.

#### 3.4.2 Protein function enrichment

**Figure 8:**
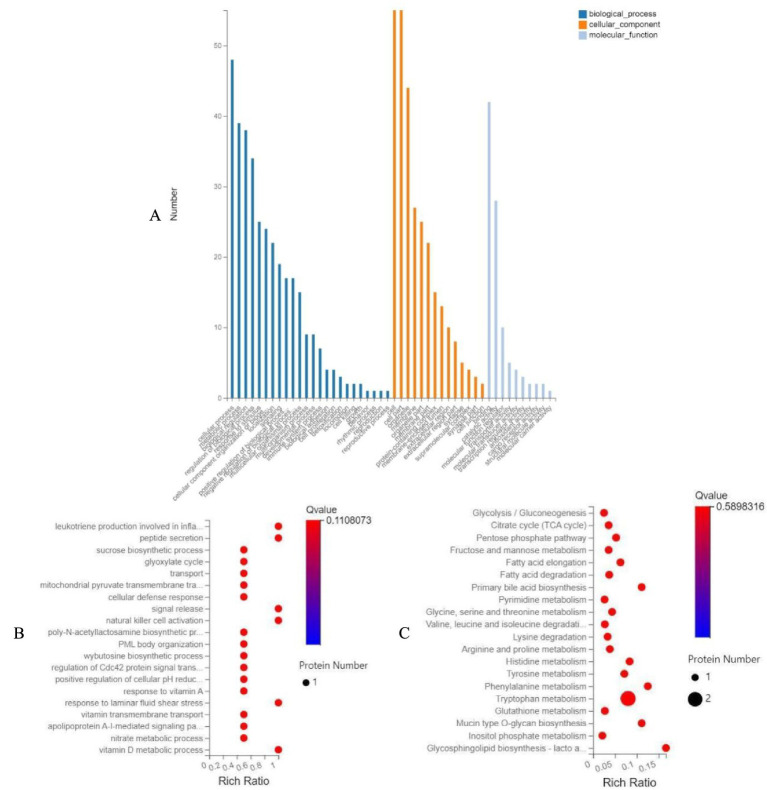
Functional enrichment analysis of differentially expressed proteins. **(A)** Gene ontology (GO) classification, **(B)** GO biological process enrichment, and **(C)** KEGG pathway enrichment analysis of differentially expressed proteins between CG and CON.

Functional annotation and pathway enrichment analyses were performed using Gene Ontology (GO) and Kyoto Encyclopedia of Genes and Genomes (KEGG) databases. GO enrichment analysis systematically annotated gene products from three functional categories: biological processes (BP), cellular components (CC), and molecular functions (MF). Significant differential expressed proteins (*p* < 0.05) were selected for enrichment analysis (Figure 8A). Under dexamethasone-induced oxidative stress, the combination group demonstrated significant upregulation of PRDX1, a key antioxidant protein, particularly associated with biological process such as natural killer cell activation. Previous studies have well-documented the antioxidant pathway mechanisms of PRDX1 (Figure 8B).

The KEGG database was employed to systematically analyze metabolic pathways and functional characteristics of gene products in cells. Based on the KEGG enrichment bubble plot (Figure 8C), DEPs were primarily enriched in the following metabolic pathways: fatty acid elongation, fatty acid degradation, glutathione metabolism, and fructose/mannose metabolism. These findings suggest that the combination treatment may regulate oxidative stress response through multiple metabolic pathways, with glutathione metabolism playing a particularly important role in cellular antioxidant defense mechanisms.

### 3.5 Effects of isochlorogenic acid combination on intestinal microflora of broilers

#### 3.5.1 Analysis of intestinal microbial diversity

**Figure 9:**
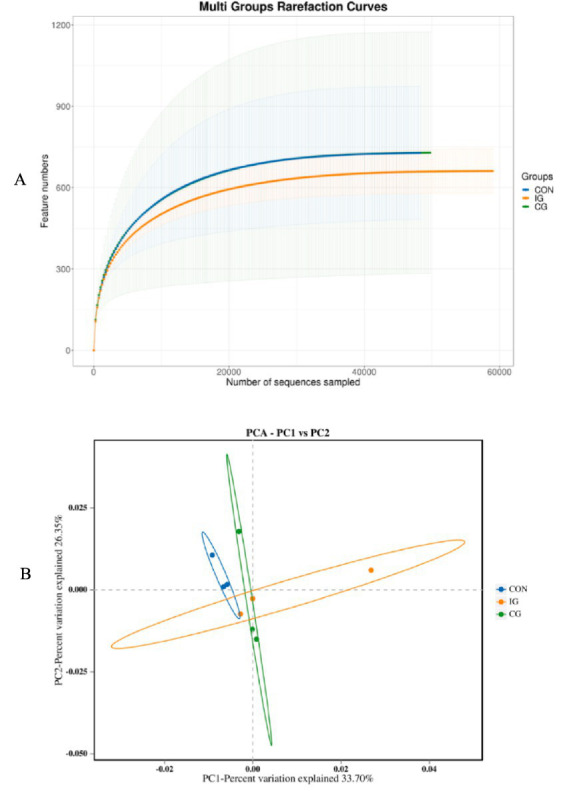
Analysis of alpha and beta diversity of intestinal microbiota in broilers among CON, IG, and CG groups. **(A)** Rarefaction curves and **(B)** principal component analysis (PCA) based on standardized Euclidean distances.

Rarefaction curve analysis was used to evaluate sequencing depth and data adequacy. A sharp increase in the curve indicates that the sequencing volume is insufficient and the number of sequence strips needs to be increased; on the contrary, it indicates that the sample sequence is sufficient, and the data can be analyzed. As sequencing depth increased, the dilution curve exhibits a plateau, demonstrating adequate sequence coverage (Figure 9A). Beta diversity analysis revealed a significant difference in the composition of the microbial community among samples. Principal component analysis (PCA) (Figure 9B) showed a distinct intra-group clustering of samples in each group, indicating a similar microbial community structure within the same group; additionally, the closer the distance between two samples, the more similar their microbial community compositions were. Samples from the injury group were somewhat separated from those of the control group and the combination group in the confidence ellipses, suggesting that there were certain differences in the intestinal microbiota among the three groups. In comparison with the control group, the samples in the combination group exhibited a higher degree of intra-group dispersion, which indicated the presence of individual differences in the intestinal microbiota among birds within the combination group.

#### 3.5.2 Intestinal microbial composition and difference analysis

**Figure 10:**
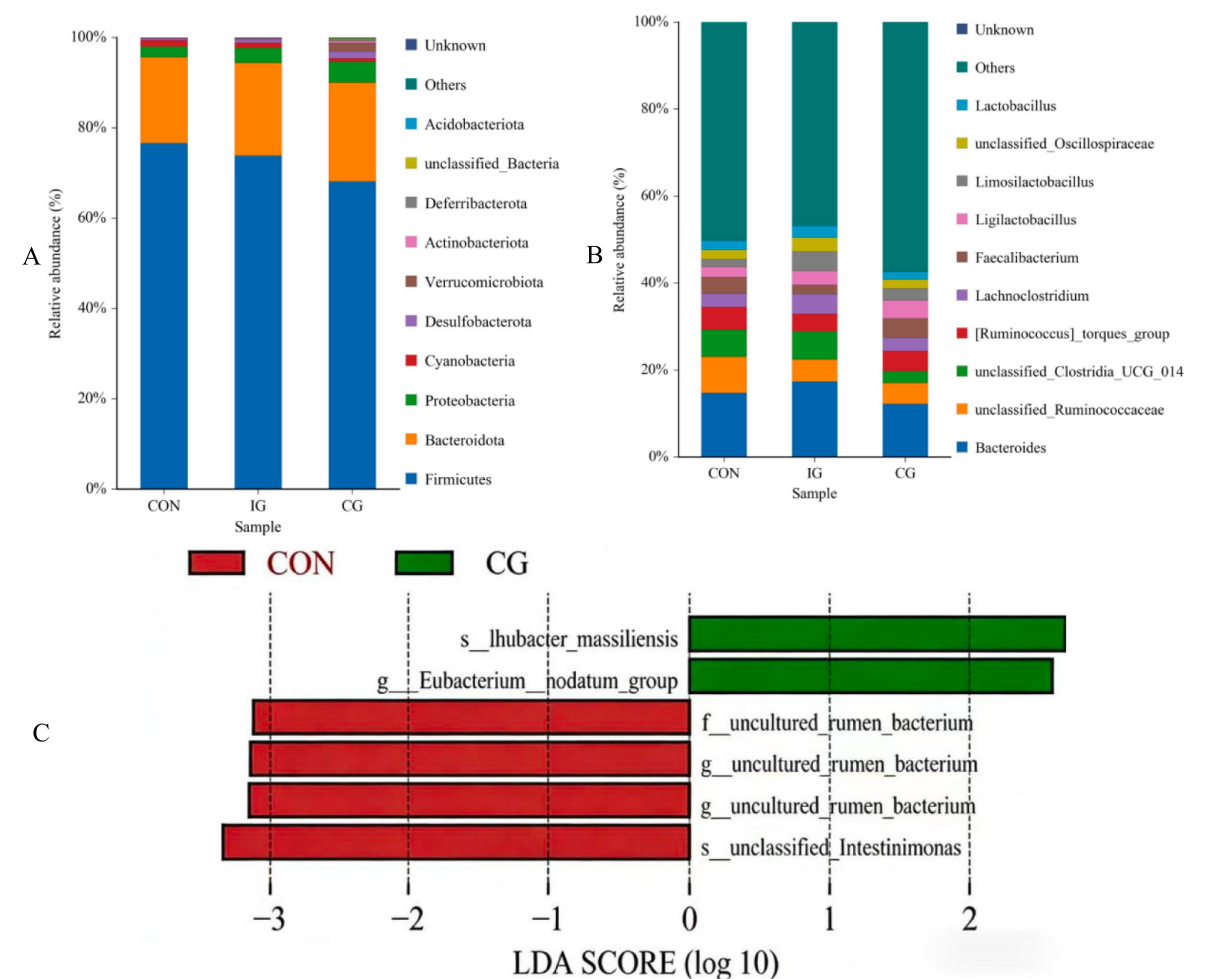
Composition and differential analysis of intestinal microbiota in broilers. **(A)** Relative abundance of microbial taxa at the phylum level, **(B)** genus-level composition, and **(C)** LEfSe analysis of differentially abundant taxa.

Statistical analysis was performed on the top 10 microbial taxa with the highest abundance in the intestinal tract of broilers. At the phylum level (Figure 10A), microbial taxa with relative abundance greater than 0.001% were analyzed. Bacteroidota, Firmicutes, and Proteobacteria were identified as the dominant phyla, collectively accounting for more than 90% of the total relative abundance. Compared with the control group, the relative abundance of Firmicutes decreased in both the combination and injury groups, whereas the relative abundances of Bacteroidota and Proteobacteria were higher in the combination group. These results indicated that the combination of isochlorogenic acid and quercetagetin exerted an effect on the intestinal microbial diversity of broilers.

At the genus level (Figure 10B), the dominant microbial taxa (top 10 in relative abundance) included Bacteroides, Clostridia_UCG-014, Oscillospiraceae, Ruminococcaceae, [Ruminococcus]_torques group, Lachnoclostridium, Faecalibacterium, Lactobacillus, Ligilactobacillus and Limosilactobacillus. In comparison with the control group, the combination group exhibited significantly higher relative abundances of Faecalibacterium and Lactobacillus (*p* < 0.05), which suggested that the combination of isochlorogenic acid and quercetagetin could promote the colonization of beneficial microbiota in the intestinal tract of broilers.

Linear discriminant analysis effect size (LEfSe) identified six taxa with significant differences between the control and combination groups (Figure 10C). Four taxa, including Intestinimonas and uncultured rumen bacteria, were significantly enriched in the control group, whereas two taxa, namely *Eubacterium nodatum* and Ihubacter massiliensis, were significantly enriched in the combination group.

## 4 Discussion

### 4.1 Effects of isochlorogenic acid combination on antioxidant properties of broilers

In poultry production systems, intestinal function represents the central interface between dietary inputs and systemic physiological outcomes. From a nutritional physiology perspective, oxidative stress should be regarded not merely as a pathological phenomenon but as a major constraint on nutrient utilization efficiency. Excess reactive oxygen species (ROS) increase maintenance energy expenditure, impair epithelial turnover, and reduce absorptive capacity, ultimately compromising feed efficiency (21). When animals are exposed to pathogens or enter a sub-health state, the balance between oxidation and antioxidation is disrupted. The decrease in the antioxidant capacity of the body and the excessive accumulation of oxygen free radicals will lead to oxidative stress in the body (22), resulting in the imbalance of reactive oxygen species content and endogenous antioxidant capacity in the body, as well as cell and tissue damage, thereby affecting the health of the animal body and the quality of livestock products (23). Malondialdehyde (MDA), a metabolic product of fatty acid peroxides, whose content changes can directly reflect the degree of oxidative damage in the organism. Particularly in metabolically active tissues or those with critical barrier functions such as the liver and intestines, MDA content is widely recognized as a core indicator for evaluating tissue oxidative damage. A higher MDA content indicates more severe lipid peroxidation and more significant tissue damage caused by oxidative stress (24). Catalase (CAT), superoxide dismutase (SOD), and glutathione (GSH) constitute the core components of the endogenous antioxidant defense system. SOD can convert excessive superoxide anion radicals in the organism into hydrogen peroxide, while CAT further decomposes hydrogen peroxide into harmless water and oxygen. As a non-enzymatic antioxidant, GSH not only directly scavenge ROS, such as hydroxyl radicals, but also participate in maintaining the activity of antioxidant enzymes, including CAT and SOD. The synergistic effect of these three components constitutes an important line of defense for the organism against oxidative stress, and their activity levels directly determine the antioxidant capacity of the organism.

In the present study, a dexamethasone (DEX)-induced oxidative stress injury model was established in broilers. Compared with the control group, the injury group exhibited significantly increased MDA levels in both serum and intestinal mucosa, accompanied by a significant reduction in serum CAT activity. These findings are consistent with the established mechanism of DEX-induced oxidative stress. As a synthetic glucocorticoid, DEX can inhibit the transcriptional expression of antioxidant enzyme genes in the organism and promote ROS generation, thereby disrupting the oxidant-antioxidant balance. This disruption further induces enhanced lipid peroxidation (elevated MDA) and decreased antioxidant enzyme activity (reduced CAT), confirming that a single injection of 3 mg/kg DEX effectively induces oxidative stress and tissue damage in broilers, particularly in the intestinal mucosa, a highly sensitive tissue.

To counteract DEX-induced oxidative stress, this study evaluated the effects of a 1:1 combination of isochlorogenic acid and quercetagetin. Compared with the injury group, the combination treatment significantly increased serum CAT, SOD, and GSH activities, reduced MDA levels in the intestinal mucosa, and enhanced CAT and GSH activities in intestinal tissue. The restoration of SOD, CAT, and GSH activities suggests that ICA + QG supplementation may alleviate oxidative stress and reduce nutrient loss under stress conditions. In broilers, oxidative imbalance diverts amino acids and energy toward stress-response pathways rather than growth-related protein accretion. Therefore, improved antioxidant buffering capacity may contribute to more efficient nutrient partitioning and maintenance of intestinal absorptive function. Notably, isochlorogenic acid, as a phenolic acid compound, possesses potent free radical-scavenging properties and can modulate the transcriptional expression of antioxidant enzyme genes. Quercetagetin, a flavonoid, also exhibits prominent antioxidant activity.

Further comparison with the control group revealed that serum SOD and GSH activities, as well as intestinal GSH activity, remained significantly higher in the combination group. In contrast, although the intestinal mucosal MDA content decreased and CAT activity increased, these changes did not reach statistical significance. This finding suggests that the 1:1 combination of isochlorogenic acid and quercetagetin not only effectively ameliorates DEX-induced oxidative stress injury but also further enhances the basal antioxidant reserve capacity of broilers under normal physiological conditions. Additionally, the coordinated changes in multiple antioxidant indices in both serum and intestinal mucosa indicate an overall improvement in redox status. However, the underlying regulatory mechanisms among these antioxidant components warrant further investigation (25).

### 4.2 Effects of isochlorogenic acid combination on serum immune performance of broilers

Immune function serves as the core defense line for maintaining the health of animal organisms, and its integrity directly determines the body’s ability to resist pathogen invasion, clear damaged cells, and maintain internal environmental homeostasis. The immune status of animal organisms is mainly determined by the functional states of two core systems: humoral immunity and cellular immunity. Globulin levels serve as key indicators of humoral immune function, whereas inflammatory cytokines reflect the activation status and regulatory balance of cellular immunity (26, 27). Complement C3 is a critical complement protein located at the junction of all complement activation pathways (28). Intestinal health is closely associated with immune homeostasis and inflammatory balance. The intestinal mucosa serves not only as a digestive and absorptive interface but also as a critical immunological barrier that integrates epithelial cells, immune cells, microbiota, and soluble mediators. Under stress conditions, such as dexamethasone (DEX) exposure, disruption of redox homeostasis is often accompanied by dysregulation of mucosal immunity and inflammatory signaling pathways.

Glucocorticoids such as DEX are well known to suppress immune cell proliferation and cytokine production, partly through modulation of innate immune signaling cascades including the Toll-like receptor (TLR)–myeloid differentiation primary response protein 88 (MyD88)–NF-κB pathway (29). In addition, excessive oxidative stress may further activate NF-κB signaling, leading to imbalanced production of pro- and anti-inflammatory cytokines. Therefore, oxidative stress and intestinal immune dysfunction are mechanistically interconnected processes.

In the present study, the combined supplementation of isochlorogenic acid and quercetagetin significantly increased serum IgM and complement C3 levels, indicating an enhancement of humoral immune responsiveness. As a central mediator of innate immunity, complement C3 bridges innate and adaptive immune responses through processes such as opsonization and immune complex clearance. The elevation of IgM, the primary antibody involved in early immune defense, indicates improved B-cell activation and primary immune surveillance capacity (30). Previous studies have reported similar findings; for instance, dietary supplementation with organic acids increased serum IgG levels in broilers (31), while composite organic acids elevated IgM and IgG levels in piglets (32). These findings are consistent with the results of the present study.

Inflammatory cytokines act as key signaling molecules mediating communication between immune cells, and their balanced expression is essential for maintaining immune homeostasis. Pro-inflammatory cytokines can activate immune cells, recruit inflammatory cells, and initiate inflammatory responses to eliminate pathogens and damaged cells; anti-inflammatory cytokines, on the other hand, inhibit the excessive secretion of pro-inflammatory cytokines to avoid secondary damage to the organism caused by uncontrolled inflammatory responses (33).

In the present study, serum IL-10 levels were significantly decreased in the injury group compared with the control group. In the combination group, serum IL-1β and IL-4 levels were significantly increased, TNF-α content showed an increasing trend without statistical significance, and IL-10 remained significantly decreased. Compared with the injury group, IL-4 and TNF-α levels were significantly elevated in the combination group. The observed upregulation of IL-1β and TNF-α in the combination group may reflect restoration of immune responsiveness under DEX-induced immunosuppression rather than excessive inflammatory activation. Controlled production of pro-inflammatory cytokines is essential for initiating effective immune defense and maintaining mucosal integrity (34). Furthermore, the increase in IL-4 may promote B-cell activation and antibody production, thereby facilitating coordination between humoral and cellular immune responses. The sustained decrease in IL-10 may be an adaptive regulation of the body in response to immune stress, that is, the combination maintains an appropriate inflammatory state by inhibiting excessive anti-inflammatory responses to ensure immune clearance efficiency and avoid immune paralysis caused by excessive anti-inflammatory effects. Nevertheless, although the results indicate modulation of systemic immune parameters, the precise regulation of intestinal innate immune signaling pathways remains to be clarified.

Overall, the isochlorogenic acid and quercetagetin combination improved immune function and enhanced cellular immunity in broilers. The underlying mechanism may be attributed to phenolic acids and flavonoids in isochlorogenic acid, which possess anti-inflammatory, antibacterial, and antiviral properties, thereby inhibiting pathogen colonization and consequently strengthening host immunity (35).

### 4.3 Effects of isochlorogenic acid combination on intestinal morphology of broilers

The small intestine is the primary site for nutrient digestion and absorption in broilers. Intestinal villus length and crypt depth of the small intestine are important indicators to measure the absorption function of the small intestine. Increased villus length reflects an expansion of the absorptive surface area and enhanced nutrient uptake, whereas reduced crypt depth indicates accelerated epithelial cell maturation and improved secretory function. Accordingly, a higher villus height-to-crypt depth ratio is generally associated with improved intestinal absorptive efficiency and a more intact mucosal structure (36).

The present results indicated that after the organism suffered from dexamethasone (DEX)-induced oxidative stress injury, the villus lengths of the ileum, jejunum, and duodenum were all shortened. These morphological alterations are of clear biological relevance. The observed villus shortening across all three intestinal segments in the injury group would reduce the total absorptive surface area by a comparable magnitude, thereby directly compromising nutrient uptake capacity. Given that the small intestine is the principal site of nutrient uptake in broilers, such structural damage may ultimately compromise feed efficiency and growth performance, which are critical economic traits in poultry production. The underlying mechanisms are as follows: excessive reactive oxygen species (ROS) induced by DEX can attack intestinal mucosal epithelial cells, destroy the cytoskeleton structure, lead to increased apoptosis and accelerated shedding of intestinal villus epithelial cells, and thereby result in shortened villus lengths. Meanwhile, oxidative stress can activate intestinal inflammatory signaling pathways, trigger intestinal mucosal inflammatory responses, further exacerbate intestinal morphological damage, and may adversely affect nutrient absorption capacity (37). On one hand, isochlorogenic acid (a phenolic acid substance) and quercetagetin (a flavonoid substance) in the combination possess significant antioxidant and anti-inflammatory activities. They can scavenge excessive ROS in the intestines, inhibit the damage of oxidative stress to intestinal villus epithelial cells, reduce cell apoptosis and shedding, thereby maintaining the villus length. In addition, these two components can inhibit the excessive secretion of intestinal inflammatory factors, alleviate intestinal mucosal inflammatory responses, and provide a favorable internal environment for the repair and development of intestinal mucosa. On the other hand, the combination may regulate the balance between proliferation and differentiation of intestinal epithelial cells, promote epithelial renewal and improve villus structural development, increase the number of villi per unit area with regular arrangement, further expand the intestinal absorption surface area, and provide a structural basis for the efficient absorption of nutrients.

Previous studies support these findings. For example, Giannenas et al. (38) found that the addition of organic acids to the feed can significantly increase the villus length and villus to villus ratio of the ileum of broilers. Consistent with these observations, the present study suggests that dietary supplementation with a 1:1 combination of isochlorogenic acid and quercetagetin promotes intestinal development, enhances digestive and absorptive functions, and may consequently improve production performance in broilers.

### 4.4 Role of the PRDX1-Nrf2-GSH pathway in dexamethasone-induced oxidative stress in broilers

Using TMT-based quantitative proteomics, PRDX1 was identified as a differentially expressed protein associated with antioxidative processes and intestinal health. PRDX1 (Peroxiredoxin-1) is a key intracellular antioxidant enzyme predominantly located in the cytoplasm, nucleus, mitochondrial matrix, and peroxisomes. Under reactive oxygen species (ROS)-mediated stress conditions, PRDX1 regulates oxidative stress either through peroxide-dependent oxidation and thiol-dependent reduction mechanisms or by functioning as a molecular chaperone. Upregulation of PRDX1 is closely associated with enhanced cellular antioxidant capacity. Previous studies have demonstrated that PRDX1 exerts protective effects in various pathological conditions by participating in the regulation of multiple ROS-related signaling pathways (39), and it may interact with key antioxidant regulatory pathways such as Nrf2.

Notably, glutathione metabolism plays a central role in cellular redox defense. In the present study, under DEX-induced oxidative stress, serum and intestinal CAT activity as well as intestinal mucosal GSH levels were markedly reduced, whereas the MDA contents in serum and intestinal mucosa were significantly increased. Combined with the proteomic findings, we speculate that the combined supplementation of isochlorogenic acid and quercetagetin may be associated with enhanced antioxidant capacity, potentially through PRDX1 upregulation and modulation of intracellular antioxidant-related pathways—including the Nrf2 signaling pathway—thereby promoting the expression of downstream antioxidant enzymes such as HO-1 and facilitating GSH synthesis. Such coordinated regulation may contribute to enhanced ROS scavenging and the maintenance of redox homeostasis.

The Nrf2 signaling pathway is widely recognized as a major antioxidant defense pathway. Previous evidence suggests that inhibition of Nrf2 transcriptional activity may exacerbate oxidative stress and inflammatory responses (40, 41). Canonically, Nrf2 remains inactive through binding to Keap1 under basal conditions; however, in response to oxidative stress, Nrf2 dissociates from Keap1, becomes activated, and translocates into the nucleus, where it initiates the transcription of downstream target genes (e.g., HO-1, NQO1) to protect against oxidative damage (42). Although the present study observed PRDX1 upregulation alongside improvements in antioxidant parameters, PRDX1 expression can also be regulated by other pathways, including NF-κB and p53. Therefore, the current evidence is insufficient to conclusively determine that the compound specifically activated the Nrf2 pathway.

Taken together, these findings suggest that the combined supplementation of isochlorogenic acid and quercetagetin alleviates DEX-induced oxidative stress and is associated with alterations in antioxidant-related proteins and glutathione metabolism. However, the current data do not allow definitive conclusions regarding specific signaling pathways, and further targeted mechanistic studies are required.

### 4.5 Effects of isochlorogenic acid combination on intestinal microflora of broilers

The intestinal microflora constitutes a significant component of the gastrointestinal ecosystem, and its structural stability and diversity are critical for maintaining tissue and organ development, immune function, metabolic processes, nutrient absorption, and overall host homeostasis (43). The intestinal microbiota not only sustain the normal morphological development of intestinal tissues and organs by modulating the proliferation and differentiation of intestinal epithelial cells but also shape the function of the host’s immune system by participating in the maturation of immune cells and regulating the secretion of cytokines. Moreover, it plays a key role in nutrient degradation and transformation, as well as vitamin synthesis, thereby directly influencing nutrient utilization efficiency and metabolic balance in the host.

In microbial diversity analysis, rarefaction curve reflects the species diversity of the microbiota and indirectly indicates the species richness within the samples. When the curve approaches a plateau, it suggests that the sequencing depth is adequate to cover the majority of species in the samples, and subsequent sequencing is unlikely to uncover new species. At this juncture, the sequencing data can be employed for accurate analysis of microbiota diversity. Principal component analysis (PCA) in the present study indicated that supplementation with a 1:1 combination of isochlorogenic acid and quercetagetin altered the intestinal microbiota structure in broilers. The potential mechanism of action may be as follows: isochlorogenic acid possesses antibacterial, antifungal, and antiviral properties, which can inhibit harmful bacteria in the intestine, thereby altering the microbial system; quercetagetin, as a flavonoid compound, can enhance the antibacterial activity of isochlorogenic acid and offer nutritional support for the growth of beneficial bacteria. The combined supplementation may contribute to partial restoration of microbiota balance disrupted by dexamethasone (DEX)-induced oxidative stress injury; however, whether this reflects a synergistic interaction between the two compounds remains to be determined.

Analysis of microbial composition showed that Bacteroidota and Firmicutes were the dominant phyla in the broiler intestine, which is consistent with previous reports (44). At the genus level, the relative abundances of fecal bacteria and Ligilactobacillus were increased in the combination group. In poultry nutrition, gut microbiota functions as a metabolic organ influencing short-chain fatty acid production, amino acid fermentation, and bile acid metabolism. The increased abundance of fecal bacteria and Ligilactobacillus observed in the combination group may enhance butyrate production, which serves as an energy substrate for enterocytes and supports epithelial integrity (45). Fecal bacteria are generally considered beneficial, as they can help maintain microbial balance by promoting the growth of commensal bacteria and inhibiting pathogenic species, while also contributing to the synthesis of vitamins and digestive enzymes. Similarly, Ligilactobacillus is recognized as a beneficial genus that can colonize the intestine, limit the proliferation of harmful bacteria, and support microbial homeostasis. It may also promote the secretion of digestive enzymes in the pancreas and small intestine of broilers and improve the digestion and absorption of nutrients.

Notably, LEfSe analysis identified *Eubacterium nodatum* as a significantly enriched taxon in the combination group. Although originally described as an anaerobic bacterium isolated from periodontal lesions, members of the genus Eubacterium are widely distributed in the gastrointestinal tract and exhibit substantial metabolic versatility. Some Eubacterium species participate in amino acid fermentation and short-chain fatty acid (SCFA) production, particularly butyrate or butyrate-related metabolites, which play essential roles in maintaining intestinal epithelial integrity, regulating mucosal immunity, and suppressing inflammatory signaling pathways (46). The enrichment of *Eubacterium nodatum* in the combination group may therefore reflect a microbiota shift toward enhanced fermentative metabolism and improved redox homeostasis. SCFAs, especially butyrate, are known to serve as energy substrates for colonocytes, reinforce tight junction integrity, and modulate host antioxidant defense via activation of intracellular signaling pathways such as Nrf2 and inhibition of NF-κB-mediated inflammation. Consequently, the increased abundance of this taxon may partially contribute to the observed improvements in intestinal morphology, antioxidant enzyme activity, and immune regulation.

In addition, the enrichment of *Eubacterium nodatum* may indicate a restructuring of anaerobic ecological niches under isochlorogenic acid and quercetagetin supplementation, potentially driven by altered intestinal pH, substrate availability, or phenolic compound metabolism. Polyphenols and organic acids are known to selectively modulate gut microbial composition by inhibiting opportunistic pathogens while promoting beneficial anaerobic fermenters (47). Therefore, the observed microbial shift suggests that the protective effects of ICA and QG may be mediated not only through direct antioxidant activity but also via microbiota-dependent metabolic regulation.

## 5 Conclusion

This study concludes that dietary supplementation of broilers with an equal-ratio combination of isochlorogenic acid (ICA) and quercetagetin (QG) effectively scavenges free radicals, enhances antioxidant capacity, mitigates dexamethasone (DEX)-induced oxidative stress damage, improves intestinal morphological structure, increases the villus-to-crypt ratio, modulates intestinal microbial balance.

In summary, combined supplementation with isochlorogenic acid and quercetagetin alleviated dexamethasone-induced oxidative stress and inflammatory responses in broiler intestinal tissues. The observed improvements were characterized by enhanced antioxidant enzyme activities, reduced lipid peroxidation, modulation of antioxidant-related proteins such as PRDX1, and partial normalization of inflammatory-associated parameters. Collectively, these findings suggest that the combined supplementation contributes to the maintenance of intestinal redox balance under glucocorticoid-induced stress conditions.

However, it is important to note that classical indicators of intestinal barrier function—including tight junction proteins (e.g., ZO-1, occludin, and claudins), epithelial permeability markers, and structural assessments of epithelial integrity—were not evaluated in the present study. Therefore, conclusions regarding intestinal barrier function or epithelial protection cannot be drawn from the current data. In addition, the absence of single-compound treatment groups limits the ability to determine whether additive or synergistic interactions exist between ICA and QGs.

Future studies incorporating barrier-specific molecular markers, permeability assays, and mechanistic pathway analyses will be necessary to further elucidate the underlying regulatory mechanisms and to determine whether the biochemical improvements observed translate into structural or functional protection of the intestinal epithelium.

## Data Availability

The original contributions presented in the study are included in the article/supplementary material, further inquiries can be directed to the corresponding authors.
